# Supplementary material for: Influenza A(H5N8) vaccine induces humoral and cell-mediated immunity against highly pathogenic avian influenza clade 2.3.4.4b A(H5N1) viruses in at-risk individuals
Source: Nat Microbiol. 2025 Dec 5;11(1):155–68. doi: 10.1038/s41564-025-02183-5 (PMC12768969; doi:10.1038/s41564-025-02183-5)
Supplement: Supplementary file 1 — Study Protocol 1. [file 41564_2025_2183_MOESM1_ESM.pdf]

nature microbiology

Supplementary information

https://doi.org/10.1038/s41564-025-02183-5

Influenza A(H5N8) vaccine induces humoral and cell-mediated immunity against highly pathogenic avian influenza clade 2.3.4.4b A(H5N1) viruses in at-risk individuals

In the format provided by the authors and unedited

Avian and seasonal influenza vaccine induced immune responses

Version 3.0, 28 October 2024

Name in Finnish: Lintuinfluenssa- ja kausi-influenssarokotteen aikaansaamat immuunivasteet

Short name: Avian influenza vaccine immunity

Short name in Finnish: Lintuinfluenssarokotteiden immuunivasteet

Code name: Avian_inf_vacc_THL2023

EU CT number: 2023-509178-44-00

THL case number: *(for THL administration)*

Version history

Version number	Date	Summary of changes
1.0	12.12.2023	First version
2.0	21.2.2024	Because of the Request for Information by the Authority<br>1. An additional secondary objective 6 has been added, and accordingly, text has been revised in sections 'Introduction', 'Secondary response variables', 'Laboratory methods' and 'Data collection from registers and participants'.<br>2. Exclusion criteria have been added<br>Typographic errors have been corrected
3.0	28.10.2024	1. Current information on the avian influenza disease, vaccinations and target groups have been updated<br>2. Sampling time window (visit 1) changed<br>3. Validation of the laboratory methods for influenza and avian influenza has been added as an exploratory objective, with additional serum samples collected for validation purposes<br>4. New information about the funding has been added

Investigator, sponsor and data controller: Finnish Institute for Health and Welfare (THL)

Principal investigator

Hanna Nohynek, MD, PhD, Professor, chief physician, Infectious Disease Control and Vaccinations Unit, Finnish Institute for Health and Welfare (THL), Mannerheimintie 166, Helsinki

Research group at THL

Collaborators outside of THL

Table of contents

Abstract 4
Introduction 5
Seroconversion 7
Seroprotection 7
Objectives 8
  Secondary objectives 8
  Exploratory objective 9
Study hypothesis 9
Study design 10
Investigational products 10
Study participants 10
Recruitment of study subjects 11
  Informed Consent 11
Inclusion criteria for cohorts 1 and 2 12
Exclusion criteria for cohorts 1 and 2 12
Study samples 12
  Sample size 13
Laboratory methods 13
  Assessment of humoral immunity against influenza 13
  Assessment of cellular immunity against influenza 14
Data collection from registers and participants (Cohorts 1 and 2) 14
Safety monitoring 14
Quality management 15
Ethical considerations 15
Data protection 16
Timing 16
Reporting of the results 16
Funding 16
References 17

Abstract

This study was designed to be carried out in an exceptional situation where highly pathogenic avian influenza A(H5N1) virus belonging to clade 2.3.4.4b, enzootic in many parts of the world including Europe, was causing large outbreaks among farmed foxes, minks and raccoon dogs in Finland in 2023. The widespread infections observed in mammals, and especially transmission of the virus between mammals, is a significant cause of concern because of the well-recognized pandemic risk of the virus. Since the beginning of 2024, the outbreaks have been controlled in Finland, and only one A(H5N1) infected bird has been detected by October 2024. Globally, however, A(H5N1) outbreaks continue occurring, and the virus has been detected in new species of mammals, i.e cows in the USA. In Europe, 75 highly pathogenic A(H5N1) and A(H7) virus detections were reported in domestic (16) and wild (59) birds between 15 June and 20 September 2024 [1]. The risk of infection with currently circulating avian A(H5) influenza viruses of clade 2.3.4.4b has been estimated to remain low for the general public in the European Union/European Economic Area (EU/EEA), but the risk of infection is low-to-moderate for those occupationally or otherwise exposed to infected animals or contaminated environments [1].

One of the planned precaution measures is to provide avian and seasonal influenza vaccines to people at risk through direct exposure to infected animals or indirectly through handling potentially infectious material. These groups include people working in fur farms, people working with poultry, official veterinarians, laboratory workers who work with avian influenza samples, people taking part in culling and disposal of affected animals and other people at special risk of avian influenza such as bird ringers.

No vaccines are administered in this study, but the vaccines distributed in the national influenza vaccination program and in the national avian influenza vaccination campaign will be evaluated. Seasonal influenza vaccination has been recommended to fur farm workers and other risk groups (from 12 September 2023 on) as part of the national vaccination program since September 2023. Based on previously reported immunological research results, it is possible to achieve partial cross-protection with seasonal influenza vaccines, although there is little evidence of its clinical significance in humans. The seasonal influenza vaccine is also considered to be beneficial by reducing the risk of co-infection with seasonal and avian influenza viruses and thereby minimizing the risk of reassortment. The updated avian influenza virus vaccine customized to match the clade 2.3.4.4b virus has been offered to the same target groups since the vaccine received market authorization and became available in June 2024.

For this clinical trial, we aim to recruit 300 subjects for assessment of avian influenza vaccine induced humoral immune responses in serum samples. From a smaller number of subjects, we will also evaluate vaccine induced mucosal immunity (salivary samples) and cell-mediated immunity from blood cell samples. The samples are to be collected before avian influenza vaccination, 3 weeks after the administration of each of the two vaccine doses and 6 and 12 months after administration of the last vaccine dose. Yearly follow-up samples and samples after possible vaccine booster doses can be collected up to 10 years.

The main aim of this study is to evaluate the immune response produced by the avian influenza vaccine, customized specifically against the H5N1 clade 2.3.4.4b. We will also evaluate the immune responses produced by the avian influenza vaccine against different H5N1 clades that have been circulating in Europe before 2023 and which will be circulating during the follow-up.

In addition, we will assess potential cross-recognition of avian influenza viruses by samples collected from subjects immunized with seasonal influenza vaccines. We will measure the immunity produced

by seasonal influenza vaccinations against different H5N1 clade viruses from serum samples collected from healthcare personnel belonging to the target group of the seasonal influenza vaccine as part of a separate clinical trial, where pre- and post-vaccination samples have been collected during each epidemic season since 2017 ("Immune Responses to influenza vaccinations and viruses among health care personnel", ClinicalTrials.gov Identifier: NCT03323112).

The distribution of seasonal influenza vaccines took place from October through November 2023, which was 1-3 months before the expected start of the distribution of avian influenza vaccines. In reality, due to delays in the licensure process, the avian influenza vaccination started only in July 2024. The avian influenza vaccine is given as a series of two doses. We will also evaluate whether receiving the seasonal influenza vaccine prior to the avian influenza vaccine, or the length of the interval between the vaccine doses influence vaccine-induced immune responses.

A small target group of laboratory personnel has previously been given avian influenza vaccines in 2009 and 2011 in Finland. Some of the individuals who previously received the vaccine are also included in the target group of the avian influenza vaccine that is now being distributed. One of the goals of this study is also to evaluate vaccine-induced responses against different virus lineages in persons who have previously received the avian influenza vaccine. We will also evaluate the cross-reactivity of antibodies, especially against the H5N1 2.3.4.4b clade, produced by the previous avian influenza vaccines from samples collected from these subjects previously, and at baseline for the current study.

For the analysis of the serum samples, we will use an in-house microneutralization test that we have developed and used in previous studies of avian influenza vaccine responses [5] and, depending on the virus clade under investigation, also the hemagglutination-inhibition (HI) assay. We will isolate peripheral blood monocytes and study vaccine induced cell-mediated immunity by analyzing the activation and persistence of avian influenza antigen induced T cell responses. Cross-recognition of T cell responses against different clade avian influenza viruses is also analyzed.

Based on the research data collected during this study, it is possible to estimate the immunity induced by the avian influenza vaccine and thus the possible level of protection in the target groups of the vaccine. Specifically, we will be able to assess the level of seroprotection and cross-protection that can be achieved with the customised vaccine product, how immunity is maintained, and on the other hand, how previous administered seasonal influenza vaccines affect the immune response. In addition, the research will be able to monitor the ability of possible new virus lineages to evade the seroprotection achieved by vaccination.

Introduction

Highly pathogenic avian influenza A(H5N1) clade 2.3.4.4b viruses have been circulating abundantly in Europe among wild and reared bird populations since 2020. As a consequence of the virus spreading from waterfowl to shorebirds, most significantly gulls in 2023, mass deaths of wild birds due to avian influenza were observed for the first time in Finland in the spring of 2023. Shortly after, infections started to spread from the wild bird populations to farmed fur animals [2]. In July 2023, highly pathogenic influenza A(H5N1) was detected in silver and blue foxes, American minks and raccoon dogs and confirmed by RT-PCR on 27 fur farms in Finland [3]. Outbreaks were detected by either RT-PCR or serology on a total of 70 farms in Finland in 2023.i). Since 2024, only one A(H5N1) virus detection in a wild bird was made in Finland. Globally, highly pathogenic avian influenza detections are being made, as well as observations of virus transmission in new mammalian species, i.e. cows in the USA.

All currently circulating highly pathogenic avian H5N1 viruses are foremost avian pathogens with a poor ability to infect and spread among humans. Human-to-human transmission is extremely rare. During the years 2021-2024, the A(H5N1) clade 2.3.4.4b virus has reportedly caused a small number of human infections globally. Although a relatively large number of people have been exposed to infected animals during the outbreak among farmed fur animals, so far, not a single human infection has been detected. In the USA, highly pathogenic influenza in cows was first reported on March 23, 2024. As of today (October 2024) per information from the USA Centers for Disease Control and Prevention, the virus has been detected in total 304 herds, in 14 states.

Previous pandemic influenza viruses have been characterized by their ability to transmit easily from one human to another and to evade prior population immunity sufficiently to allow sustained transmission. Although the population immunity to avian influenza viruses is low or non-existing, there are several factors that have prevented the transmission of the virus between humans. Most importantly, the surface glycoproteins of avian influenza viruses have a low affinity to the cell receptors in the upper respiratory tract of humans. Due to the intrinsic nature of influenza viruses mutating in response to environmental changes, there is a risk of mammalian adaptation of avian influenza viruses during replication in mammalian hosts. Whole-genome sequencing and genomic analyses of viruses isolated during the fur-farm outbreak have been studied to track changes in the virus genomes, with a focus on mutations that may have indications on the zoonotic potential of the virus. The analyses revealed several virus isolates with mutations indicative of an early mammalian adaptation. Most notably, mutations in the polymerase gene PB2 allowing for more efficient replication in the upper respiratory epithelia of mammals, seem to have evolved independently on a number of affected farms. The accumulation of such mutations among circulating avian influenza viruses may increase the pandemic potential of the virus by improving its ability to transmit and cause disease in humans. Although human transmission still appears to be very rare, spill-over events from the wild bird population to mammals are becoming more frequent, and the risk of sporadic infections in humans cannot be neglected. Altogether, sporadic and small outbreaks of avian influenza infections have been seen in more than 40 mammalian species.

Virus circulation at the human-animal interface, as is demonstrated by the ongoing fur farm outbreaks, further increases the risk of emergence of a pandemic avian influenza virus. Due to Finland's exceptional epidemic situation and the increased risk of a pandemic, Ministry of Social Affairs and Health (STM) has made a decision to participate in the EU's joint vaccine procurement. AFLUNOV, manufactured by Seqirus, has been on the market since 2010, contains the H5N1 A/turkey/Turkey/1/2005 virus, which is a virus of the development line 2.2.1 [6]. Antigenically and genetically, the older vaccine virus differs from the currently circulating development line 2.3.4.4b H5 viruses, which is why the manufacturer is currently customizing the vaccine to the A/Astrakhan/3212/2020 (H5N8) strain, in which the H5 protein is almost identical to the H5 protein of circulating avian strains found in Finland. It is expected that a tailored vaccine would provide moderate to good protection against the circulating clade 2.3.4.4b viruses through cross-reactivity and protection. Cross-protection can be based on both antibody-mediated and cell-mediated immunity, which recognizes common structures of the vaccine virus and circulating viruses. However, it must be noted that influenza viruses are constantly changing as the virus multiplies, but also through reorganization of the virus's inheritance, which may result in a virus capable of circumventing the protection provided by the vaccine.

The vaccines were expected to be in use in the beginning of 2024, but due to delays in the registration process, vaccination was initiated only in June 2024. The target groups to be vaccinated were decided based on an updated risk assessment. The target groups include people working in fur farms and poultry industry, official veterinarians and laboratory workers or people working in the collection or analysis of bird influenza samples. In addition, the vaccine is offered to other persons

over the age of 18 who are at special risk, such as bird ringers. The vaccine is given in a series of two doses, where the second dose is given three weeks or later after the first dose.

There is no approved correlate of immunity to protection against infections due to influenza A (H5N1), but it is widely believed that the protection against influenza can be conferred by serum hemagglutinin (HA) antibodies. In the licensing of pandemic influenza vaccines, assessment of seroconversion and seroprotection is a critical requirement.

In the assessment of influenza vaccines, two key immunological parameters are considered: seroconversion and seroprotection.

Seroconversion

Definition: The acquisition of detectable virus-specific antibodies in response to vaccination

Measurement: Quantification of anti-HA antibodies pre- and post-vaccination to observe an increase

Significance: The observation of seroconversion indicates that the vaccine has induced a significant increase in virus-specific antibodies that can be measured by several laboratory methods.

Seroprotection

Definition: The presence of a sufficiently high antiviral antibody levels expected to be associated with protection against a disease.

Measurement: Evaluation of anti-HA antibody levels post-vaccination to determine if they reach a protective threshold.

Significance: Seroprotection is expected if sufficiently high levels of antiviral antibodies are found in the sera of vaccinated individuals that likely gives protection against influenza disease. *Seroprotection rate* refers to the proportion of subjects in whom the level of anti-HA (or neutralizing) antibodies exceeds the value considered to be the limit of a significant level in terms of protection, e.g. the HI titer measured with the hemagglutination inhibition test (HI test) ≥1:40. In addition to this, it is possible to evaluate the seroprotection as an increase in antibody concentration when the pre-vaccination sample is seropositive (e.g. HI titer >10) and to estimate in how many of the study subjects the antibody (Ab) increase exceeds the factor considered as a critical response, a fourfold increase in the titer. In other words, if the pre-vaccination serum is negative, the postvaccination serum titer must be ≥40; if the pre-vaccination serum is positive, at least a fourfold increase in antibody titer is required.

According to the summary of product characteristics (SPC) of the currently marketed vaccine [3], based on the antibody responses elicited by the A/turkey/Turkey/1/2005 vaccine strain after two doses of AFLUNOV, a protective response may not be elicited in all recipients of the vaccine. According to previous research results presented in the SPC, the seroprotection rate against the homologous A/Vietnam/1194/2004 strain measured by the microneutralization (MN) test in adults aged 18-60 who received two doses of the vaccine was 67% (95% confidence interval 60–74) to 85% (78–90) and the seroconversion rate was 65% (58–72) to 83% (77–89). The seroprotection rate against the homologous A/turkey/Turkey/1/2005 strain measured with the MN test was 85% (79–90) and the seroconversion rate was 93% (89–96). Some cross-reactive immunity has been observed against H5N1 viruses from other clades. However, it is not known to what extent protection can be achieved against different H5N1 clade viruses. There is no immunological research data yet available for the avian influenza vaccine tailored against the A/Astrakhan/3212/2020 (H5N8) strain.

In a previously published study, it was found that the seasonal influenza vaccine produced neutralizing antibodies also against H5N1 viruses in a large proportion of the subjects, indicating the

existence of an antibody-dependent cross-reactivity [7]. At one month after seasonal influenza vaccination, 28/38 (74%) showed a >4-fold increase of neutralizing antibody titer measured with neutralization test, and 13/38 serum samples (34%) showed a 20-fold increase of neutralizing Abs against influenza H5N1. HI titers against H5N1 remained at undetectable levels after seasonal vaccination in 13/38 (34.2%) of donors [7]. Also, H5N1 virus-specific CD4 T cell responses (in small frequency) were detected in several persons at baseline, and vaccination with a seasonal influenza enhanced the frequency of H5N1 specific CD4 T cells [7]. At 1 month after seasonal influenza vaccination influenza H5N1 specific CD4 response was detectable in 41.6% of study participants. Another study reported that vaccination with seasonal influenza vaccine provided a partial protection against a lethal H5N1 challenge in mice, and it was suggested that seasonal influenza vaccination could be used as an interim measure to decrease morbidity and mortality from H5N1 prior to the availability of a specific vaccine [8]. Mouse experiments have also shown that non-neutralizing antibodies against HA epitopes produced by multiple immunizations with seasonal influenza vaccines that are at least in part directed against conserved HA epitopes, mediated protection from challenge with H5N1 even in the absence of detectable neutralizing antibodies against the H5N1 virus [9]. Based on these findings, it is possible that partial cross-reactivity, potentially even some cross-protection may be achieved with the seasonal influenza vaccine through both neutralizing and non-neutralizing antibodies, and T-cell-mediated immunity, at least in some of the vaccinated. Since the concentration of serum antibodies remains at its highest for about three months after vaccination, and since the proportion of potentially cross-reacting antibodies is relatively small, it would be expected that the effect (cross-reactivity and possible cross-protection) may last a relatively shorter time after vaccination.

The samples collected in this study are unique, because they represent immune responses against the first avian vaccinations and in a situation, where no human spread of the avian influenza has happened so far. Previous infection and vaccination history may have unknown effects on responses to later administered vaccines, such as the avian influenza vaccine under investigation in this study. Studying innate immune responses can help to better understand the immune responses triggered by vaccination that influence the development of acquired immunity. Thus, it is extremely important that a H5N1 virus-specific avian influenza vaccines are used to protect fur farm workers and other people potentially exposed to avian influenza viruses. It is also very important that when the H5N1 vaccination campaign starts in Finland we collect information on the immunogenicity, cross-reactive immune responses and the duration and specificity of humoral and cell-mediated immunity induced by avian and seasonal influenza vaccines. The data obtained in our project has huge national and international needs and interests and the information is essential for global preparedness against influenza pandemics.

Objectives

The main objective of this study is to evaluate the humoral and cell-mediated immune responses induced by the customized avian influenza vaccine, specifically against the H5N1 2.3.4.4b clade.

Secondary objectives

To evaluate

1. humoral immune responses produced by the avian influenza vaccine against different H5N1 development lineages (clades) that have occurred before 2023, or that are identified to occur during the study

2. cellular immune responses induced by the customized avian influenza vaccine, against different H5N1 clades
3. the effects of prior seasonal influenza vaccinations on the immune responses induced by the avian influenza vaccine
4. humoral immunity produced by seasonal influenza vaccinations against different H5N1 lineages evaluated from samples collected from healthcare personnel belonging to the target group of the seasonal influenza vaccine ("Immune responses to influenza vaccinations and viruses among health care personnel", ClinicalTrials.gov Identifier: NCT03323112)
5. humoral and cellular immune responses induced by the H5N1 2.3.4.4b customized vaccine in subjects who have previously received avian influenza vaccines
6. humoral and cellular immune responses induced by other respiratory or non-respiratory pathogens and vaccines against them, and their potential association with immune responses induced by seasonal and avian influenza vaccines or viruses

Exploratory objective

Development of laboratory methods for influenza and avian influenza

Primary outcome measure: Seroconversion proportion in all study subjects 3 weeks after the second dose given at least 21 days after the first dose, as assessed by the microneutralization test.

Study hypothesis

It is expected that a two-dose avian influenza vaccination schedule will induce **seroprotection** in at least 70% of the vaccinees (the lower bound of the two-sided 95% CI for the percent of subjects achieving seroconversion meets or exceeds 70%). The criterion for seroprotection is titer value ≥ 80 as analyzed by microneutralization test (MNT).

Secondary response variables:

- Rate of **seroconversion** defined as the percentage of subjects with either a pre-vaccination MNT titer < 1:10 and a post vaccination MNT titer ≥ 1:80 or a pre-vaccination MNT titer ≥ 1:10 and a minimum four-fold rise in post-vaccination HI antibody titer.
- Geometric mean titers at pre- and post-vaccination.
- Detectable increase in T cell (CD4+ and CD8+) responses against H5N1 virus specific antigens in avian influenza virus vaccinated individuals.
- Geometric mean antibody titers and detectable T responses against other pathogens and/or vaccines of interest in the target group of the avian influenza virus vaccine.

Study design

This is a prospective phase IV clinical trial for evaluating the humoral and cell-mediated immune responses in population groups to be vaccinated with the avian influenza vaccine according to the national recommendations in Finland.

The study is observational in nature as no vaccines are allocated or administered through this study protocol. However, blood and saliva samples are obtained to evaluate vaccine immunogenicity, and thus, the study is defined as an intervention study according to the EU regulation on clinical trials (536/2014). In the study, only licensed vaccines given within the framework of the national vaccination program/campaign are monitored, which have been given in accordance with the national vaccination campaign. In addition, the blood and saliva samples collected in the study do not pose more than minimal additional risk or burden to the safety of the subjects compared to normal clinical practice. Therefore, this study is a low-intervention trial. No control arm is included in this study.

Investigational products

The currently available zoonotic influenza vaccine, AFLUNOV, is based on the A/turkey/Turkey/1/2005 (H5N1) vaccine strain. The vaccine contains 7.5 µg of H5 hemagglutinin antigen and MF59C.1 adjuvant containing squalene and polysorbate. The AFLUNOV vaccine is licensed for all adults 18 years of age or older. The vaccine is given as a series of 2 doses at least 21 days apart (3 weeks) by intramuscular injections according to the marketing authorization. The manufacturer of AFLUNOV vaccine, Seqirus S.r.l., has 9.10.2023 received marketing authorization for the medicinal product Zoonotic Influenza Vaccine Seqirus, based on the same strain. Seqirus S.r.l., has applied for authorization for an updated composition, which is based on the A/Astrakhan/3212/2020 (H5N8) strain [10]. The updated zoonotic influenza vaccine [4] will be evaluated as the investigational product under this protocol. Seasonal influenza vaccine in the national vaccination program will be evaluated as well. The vaccine in the national vaccination program for the season 2023-2024 is VaxigripTetra, manufactured by Sanofi.

Study participants

The study participants will include subjects aged 18 to 65 years who belong to the target groups for avian influenza vaccination (part of which have previously received an avian influenza vaccine) or who have provided samples for evaluation the immunity against influenza and other vaccine preventable diseases.

Cohort 1

Study subjects for cohort 1 will include subjects who belong to the target groups for which the avian influenza vaccine will be recommended as part of the national H5N1 vaccination campaign (targeted sample size 300). These groups can include but are not limited to people working on fur and poultry farms, official veterinarians and laboratory personnel at THL, Finnish Food Authority, Turku University (TY), Helsinki University (HY) and Helsinki University Hospital (HUS).

Cohort 2

Some of the subjects of cohort 1 form a separate cohort 2. Study subjects for cohort 2 will include persons who previously belonged to the target group who received the H5N1 vaccine (ref. 3) in 2009 (pre-pandemic H5N1, inactivated, AS03-adjuvanted A/Indonesia/5/2005-like split virion vaccine, 3.75µg HA, GlaxoSmithKline), and/or 2011 (inactivated, adjuvant-free A/Vietnam/1203/2004-like whole virus H5N1 vaccine, 7.5µg HA, Baxter) and which also belong to the target group of the recommendation for the avian influenza vaccine.

Cohort 3

The study population (cohort 3) will include healthcare personnel who participate in a separate clinical trial on immunogenicity against influenza vaccine, where pre- and post-vaccination samples have been collected during each season since 2017 ("Immune Responses to influenza vaccinations and viruses among health care personnel", conducted by THL and HUS, ClinicalTrials.gov Identifier: NCT03323112). New samples will not be obtained from these subjects, but the samples obtained in the separate trial will be used. Influenza vaccinations of health and social care professionals are part of statutory control of infectious diseases and patient safety.

Recruitment of study subjects

To recruit persons who are potentially exposed to avian influenza virus in their work (cohort 1), THL cooperates with Finnish Food Authority and HUS. Finnish Food Authority has contact details of different target groups, like fur farmers and poultry farmers (who are asked to inform their workers), the veterinarians and laboratory personnel at Finnish Food Authority. Laboratory workers at THL and HUS, TY and HY can be contacted through these institutes (cohorts 1 and 2). The subjects in the cohort 3 will not be contacted, since the consent in the influenza trial covers the use of the samples in the current study.

If the target groups for avian influenza vaccination widen to new risk groups, means to contact the new groups will be searched. THL may also use media such as web pages, announcements and articles in local newspapers and magazines to increase awareness of the study among the population. Most fur farms are located in counties of South, Central or North Ostrobothnia, and the recruitment will focus to these areas.

THL will send recruitment material including an 1-page invitation letter, the full study information sheet and the Informed Consent Form (ICF) with study visit appointment instructions, either directly to the target groups or to the home addresses to those who have expressed interest to participate.

If the avian influenza vaccinations start so early that the study cannot recruit participants before the vaccinations, or only a small part of the target sample could be recruited before the start of avian influenza vaccinations, THL will apply for approval to identify from the national vaccination register the subjects who have already received the vaccine and send invitations to the home addresses of a random sample of these subjects, requested from the Population Information System of the Digital and Population Data Services Agency.

Informed Consent

After receiving the invitation material including the full information sheets and examples of the ICF, the subjects will be encouraged to contact the study staff to ask questions and discuss the details. They will have time to consider their participation, before they make an appointment to the study visit. The recruitment material will be available in Finnish, Swedish or English language.

Informed consent will be obtained from the subjects in cohorts 1 and 2 before any study procedures are performed. The subject must be able to read and understand the information adequately.

A consent is not needed from the subjects in the cohort 3, since the consent obtained in the trial ´Immune Responses to influenza vaccinations and viruses among health care personnel´, NCT03323112 covers the use of the samples to this purpose.

Inclusion criteria for cohorts 1 and 2

- Age 18 to 65 years
- Belongs to the target group of the avian influenza vaccine
- Is planning to receive at least one dose of the avian influenza vaccine
- Is a native speaker of Finnish, Swedish or English
- Has given written informed consent
- Home address in Finland
- Is able to give samples 3 weeks after each vaccine dose and preferably also participate in the follow-up at 6 and 12 months
- Pregnant and lactating women can participate in the study.

Exclusion criteria for cohorts 1 and 2

- Has any medical contraindications to influenza vaccination
- History of an anaphylactic (i.e. life-threatening) reaction to any of the constituents or trace residues of the vaccines (egg and chicken proteins, ovalbumin, kanamycin and neomycin sulphate, formaldehyde, hydrocortisone and cetyltrimethylammonium bromide).

Study samples

The study samples will be collected in THL´s laboratory and THL´s partners laboratory centres, primarily in HUS Diagnostic Center, Nordlab,South Ostrobothnia Welfare Region Laboratory and Fimlab. Laboratory analyses on samples are carried out at THL, or at collaborators' laboratories.

Samples will be collected at 5 study visits during the first year. A serum sample (maximum 10 ml of blood) is collected from the subjects before the 1st vaccine dose, 21 days (18-24 days) after the 1st dose and 21 days (18-24 days) after the 2nd dose. Follow-up samples are scheduled 6 months and 12 months after the last vaccine dose. In addition, a blood sample (maximum 50 ml) is taken from a small number of subjects (maximum 150) for evaluation of cell-mediated immunity. A saliva sample can also be taken from these subjects (maximum -150) for examination of mucosal immunity. Additional serum samples (up to 50 ml) will be collected from a small group of subjects (maximum of 20, distinct from those providing samples for cell-mediated immunity evaluation) to support the development of laboratory methods for influenza and avian influenza. Cell and saliva samples are taken only from those recruited in the capital area (Uusimaa county). Follow-up samples, if feasible, can be collected for up to 10 years after the last vaccine dose.

Visit number	Timing in relation to vaccination	Time window
1	0 sample	0-60 days before vaccination
2	1 mo	18-24 days after 1st vaccine dose
3	1 mo	18-24 days after 2nd vaccine dose
4	6 mo	150-210 days after 2nd vaccine dose
5	12 mo	330-390 days after 2nd vaccine dose

Follow-up samples may be collected annually for up to 10 years.

In alternative recruitment, persons who have already received the vaccine are invited to the study 18-24 days after the most recent vaccine dose (1st or 2nd dose).

Sample size

The targeted sample size of 300 was determined using a formula, as illustrated below, for estimating sample sizes for binomial proportions. This calculation was based on a desired 95% confidence level, a 5% margin of error, and an assumed seroconversion rate of 75%. The result indicates a minimum sample size of 288 subjects required to accurately estimate the proportion of subjects achieving seroconversion. With this determined sample size, the lower limit of the 95% confidence interval is equal to or exceeds 70%.

$$n = \frac{Z^2 \cdot p \cdot (1-p)}{E^2}$$

Where:

- n is the required sample size.
- Z is the Z-score corresponding to the desired confidence level.
- p is the assumed proportion of seroconversion.
- E is the margin of error."

Laboratory methods

Laboratory analyses on samples are carried out at THL or at partner laboratories in Finland, or in the European Union region or European Economic Area, or at WHO Collaborating Centre for Reference and Research on Influenza (London, UK) in which case cooperation is agreed upon in a separate agreement with THL.

Assessment of humoral immunity against influenza

We will assess antibody-mediated immunity from serum samples using various methods. The most important serological assay is the microneutralization test (MNT), which measures the functionality of the antibodies and which we have used in previous studies of avian influenza vaccine responses [5]. In a previous study conducted by THL a MNT titer ≥ 80 was found to correspond to the value of the HI test ≥ 405. Depending on the virus lineage under investigation, the functional activity of antibodies can also be assessed using the hemagglutination-inhibition (HI) assay. The concentration

of antigen-specific antibodies and antibody subclasses, as well as functional properties of the antibodies can be assessed with different methodologies.

Assessment of cellular immunity against influenza

We will isolate peripheral mononuclear cells (PBMCs) from the blood samples and study cell-mediated immunity from the samples, especially the activation of T cell immune responses activated by different influenza virus lineages or antigens. Cellular immunity will be assessed from thawed PBMC samples after stimulation with influenza viruses or virus antigens in cell culture. Cytokine (IFN-gamma and others) responses will be assessed by measuring the concentration of secreted cytokines in the cell culture media with Luminex technology. Additionally, mRNA expression of cytokines and other factors (e.g. inflammatory mediators and transcription factors) are measured from specific cell populations enriched with magnetic cell separation or sorted with FACSAria flow cytometer. The methods to analyze COVID vaccine induced CD4 and CD8 T cell responses were recently developed [12-14] and the methodology can relatively rapidly be transferred to analyze cell-mediated immunity against influenza vaccine induced immunity.

The samples will also be used to examine humoral and cellular immunity against other pathogens and vaccines of interest as well as innate immunity and inflammatory responses to these factors. The potential association of these responses with the responses against seasonal and avian influenza vaccines and viruses will be described.

The samples may also be used in the validation and development of new immunological assays.

Data collection from registers and participants (Cohorts 1 and 2)

Information of the seasonal and avian influenza vaccinations and other vaccinations is collected from the national vaccination register from the time of the study and previous 5 years (part of the Register of Primary Health Care Visits, Avohilmo), so that the effect of previous vaccinations on the measured immune responses can be evaluated. The national infectious diseases registry will be utilized to verify participants' prior exposure to seasonal or avian influenza viruses or other microbes causing infectious diseases. Subjects are asked to answer the questions on the consent form.

Safety monitoring

In the study, only licensed vaccines given within the framework of the national vaccination program/campaign are monitored, which have been given in accordance with the SPC or according to the national recommendation. Therefore, this study is a low-intervention trial.

The study subjects are requested to inform the research staff if they suspect a post-vaccination adverse event (following the zoonotic influenza vaccine) that differs from the usual local symptoms (swelling, pain or bruising at the injection site) or general symptoms (fever, muscle and headache, fatigue, irritability, malaise and chills). At every contact with the study subjects, the study staff will ask whether any potential adverse events have occurred. The study subjects are also requested to inform the study staff if they suspect adverse events related to the study procedure, which differ from the usual swelling, pain or bruising at the blood sampling site.

The investigators will evaluate whether the adverse events are serious and whether the event is related to avian influenza vaccination or any other study procedure. THL will report all serious adverse events related to study procedures and all suspected unexpected serious adverse reactions

(SUSAR) related to avian influenza vaccines to the regulatory authority within time limits required by EU regulation on clinical trials. The safety follow-up for each subject will start at enrolment and last 3 months after the last vaccine dose. For adverse events related to the study procedure, the follow-up will extend until the end of the study. Additionally, THL will provide Fimea with an annual report including all serious adverse events reported to it during the study with a short update on subject safety. Adverse events potentially related to vaccines in the Finnish national vaccination programs/campaign are primarily reported to and assessed by Fimea. If a suspected unexpected serious adverse reaction reported by a subject is unequivocally found in the copy of the adverse reaction register received by THL from Fimea, it is not reported again to Fimea.

Quality management

- quality control of the paper CRFs and forms by THL staff at/before the data entry
- searching for discrepancies and impossible values in the study database
- risk-based monitoring by the sponsor

The study is carried out, data is produced, documented and reported according to the study design, good clinical practice (GCP) and up-to-date regulatory requirements.

The sponsor has established a systematic quality assurance plan, based on a risk assessment, which includes a monitoring plan. The operation and quality assurance of the study are based on written guidelines approved by the PI.

Study documentation will be maintained in a trial master file (TMF)

The study will follow the sponsor's internal quality guidelines: data management and archiving, data protection and security, contract management, and study specific SOPs.

Informed Consent Form (ICF) will be obtained from the study participants prior to any research activities; and the identity of the subject will be verified before the consent is obtained. Informed consent forms, withdrawals and protocol deviations are recorded in the THL electronic information system. Paper CRFs and forms are verified by THL study staff before data entry.

Ethical considerations

No vaccines are given in the study. The only interventions are the additional diagnostic methods, i.e., data collection and blood sampling performed by trained professionals. Thus, the study procedures carry out only minimal risk or harm to the subjects. All subjects have given a written informed consent, as described in section ‘Informed consent’. The study protocol will be sent to the Medicines Agency (Fimea) and the National medical research ethics committee (TUKIJA) for evaluation via the EU portal (CTIS) and the study will not start before their approval.

The participant does not benefit personally from the study. However, the study results add knowledge on preventing avian influenza by vaccination, which benefits especially the risk groups participating in the study. The information is also important in the current epidemiologic situation in Finland for planning prevention and mitigation of potential worldwide pandemic avian influenza.

Data protection

THL is the controller of the study register. Data is processed in accordance with the EU Data Protection Regulation (2016/679) and the national data protection law (1050/2018). The study's data protection notice will be published on the study's website.

Timing

The study will start when avian influenza vaccinations are planned to start, in 2024. The duration of the clinical phase of the study depends on whether follow-up is deemed necessary and feasible, a minimum of 1 year and a maximum of 10 years. Analyses of the samples are started immediately at the start of the study and analysis of the samples are continued even after the end of the clinical phase. The study material will be maintained at THL for 25 years after the clinical phase of the study.

Reporting of the results

The results are reported both as scientific publications and by communicating to stakeholders and communicating in the form of bulletins. The results of this national post-marketing immunological vaccine study will play a pivotal role in informing critical decision-making processes related to public health and vaccination strategies. The gathered immunological data from this immunogenicity study will be analyzed to evaluate the vaccine's ability to generate humoral and cell-mediated immune responses and the potential protective immunity induced against different avian influenza A(H5N1) strains. Decision-makers, including health authorities and policymakers, will leverage these findings to refine vaccination policies, prioritize high-risk populations, and make informed adjustments to public health measures.

Funding

References

Ref. 1. https://www.efsa.europa.eu/en/efsajournal/pub/9057

Ref. 2 Lindh E, Lounela H, Ikonen N, Kantala T, Savolainen-Kopra C, Kauppinen A, Österlund P, Kareinen L, Katz A, Nokireki T, Jalava J, London L, Pitkäpaasi M, Vuolle J, Punto-Luoma AL, Kaarto R, Voutilainen L, Holopainen R, Kalin-Mänttäri L, Laaksonen T, Kiviranta H, Pennanen A, Helve O, Laamanen I, Melin M, Tammiranta N, Rimhanen-Finne R, Gadd T, Salminen M. Highly pathogenic avian influenza A(H5N1) virus infection on multiple fur farms in the South and Central Ostrobothnia regions of Finland, July 2023. Euro Surveill. 2023 Aug;28(31):2300400

Ref 3. Kareinen L, Tammiranta N, Kauppinen A, Zecchin B, Pastori A, Monne I, Terregino C, Giussani E, Kaarto R, Karkamo V, Lähteinen T, Lounela H, Kantala T, Laamanen I, Nokireki T, London L, Helve O, Kääriäinen S, Ikonen N, Jalava J, Kalin-Mänttäri L, Katz A, Savolainen-Kopra C, Lindh E, Sironen T, Korhonen EM, Aaltonen K, Galiano M, Fusaro A, Gadd T. Highly pathogenic avian influenza A(H5N1) virus infections on fur farms connected to mass mortalities of black-headed gulls, Finland, July to October 2023. Euro Surveill. 2024 Jun;29(25):2400063]Ref. 4. https://www.ema.europa.eu/en/documents/product-information/aflunov-epar-product-information_fi.pdf

Ref 5. Haveri A, Ikonen N, Savolainen-Kopra C, Julkunen I. Long-lasting heterologous antibody responses after sequential vaccination with A/Indonesia/5/2005 and A/Vietnam/1203/2004 pre-pandemic influenza A(H5N1) virus vaccines. Vaccine. 2021 Jan 8;39(2):402-411

Ref 6. https://www.ema.europa.eu/en/documents/scientific-guideline/influenza-vaccines-non-clinical-clinical-module_en.pdf

Ref 7. Gioia C, Castilletti C, Tempestilli M, Piacentini P, Bordi L, Chiappini R, Agrati C, Squarcione S, Ippolito G, Puro V, Capobianchi MR, Poccia F. Cross-subtype immunity against avian influenza in persons recently vaccinated for influenza. Emerg Infect Dis. 2008 Jan;14(1):121-8

Ref 8. Rockman S, Brown LE, Barr IG, Gilbertson B, Lowther S, Kachurin A, Kachurina O, Klippel J, Bodle J, Pearse M, Middleton D. Neuraminidase-inhibiting antibody is a correlate of cross-protection against lethal H5N1 influenza virus in ferrets immunized with seasonal influenza vaccine. J Virol. 2013 Mar;87(6):3053-61

Ref 9. Roos A, Roozendaal R, Theeuwsen J, Riahi S, Vaneman J, Tolboom J, Dekking L, Koudstaal W, Goudsmit J, Radošević K. Protection against H5N1 by multiple immunizations with seasonal influenza vaccine in mice is correlated with H5 cross-reactive antibodies. Vaccine. 2015 Mar 30;33(14):1739-47

Ref 10. https://www.ecdc.europa.eu/sites/default/files/documents/First-identification-human-cases-avian-influenza-A-H5N8-infection.pdf

Ref 11. Avian influenza overview June–September 2023, EFSA Journal Scientific Report, https://doi.org/10.2903/j.efsa.2023.8328

Ref 12. Hurme A, Jalkanen P, Heroum J, Liedes O, Vara S, Melin M, Teräsjärvi J, He Q, Pöysti S, Hänninen A, Oksi J, Vuorinen T, Kantele A, Tähtinen PA, Ivaska L, Kakkola L, Lempainen J, Julkunen I. Long-Lasting T Cell Responses in BNT162b2 COVID-19 mRNA Vaccinees and COVID-19 Convalescent Patients. Front Immunol. 2022 Apr 22;13:869990.

Ref 13. Belik M, Jalkanen P, Lundberg R, Reinholm A, Laine L, Väisänen E, Skön M, Tähtinen PA, Ivaska L, Pakkanen SH, Häkkinen HK, Ortamo E, Pasternack A, Ritvos MA, Naves RA, Miettinen S,

Sironen T, Vapalahti O, Ritvos O, Österlund P, Kantele A, Lempainen J, Kakkola L, Kolehmainen P, Julkunen I. Comparative analysis of COVID-19 vaccine responses and third booster dose-induced neutralizing antibodies against Delta and Omicron variants. Nat Commun. 2022 May 5;13(1):2476.

Ref 14. Obach D, Solastie A, Liedes O, Vara S, Krzyżewska-Dudek E, Brinkmann L, Haveri A, Hammer CC, Dub T, Meri S, Freitag TL, Lyytikäinen O, Melin M. Impaired immunity and high attack rates caused by SARS-CoV-2 variants among vaccinated long-term care facility residents. Immun Inflamm Dis. 2022 Sep;10(9):e679.

ChatGTP3.5 was used to describe the definition of seroconversion and seroprotection, which was reviewed and approved by experts. ChatGTP3.5 was also used in formulating the sample size calculation, after which a statistician evaluated the validity of the calculation.
