# Supplementary material for: Influenza A(H5N8) vaccine induces humoral and cell-mediated immunity against highly pathogenic avian influenza clade 2.3.4.4b A(H5N1) viruses in at-risk individuals
Source: Nat Microbiol. 2025 Dec 5;11(1):155–68. doi: 10.1038/s41564-025-02183-5 (PMC12768969; doi:10.1038/s41564-025-02183-5)
Supplement: Supplementary file 2 — Reporting Summary [file 41564_2025_2183_MOESM2_ESM.pdf]

nature portfolio

Corresponding author(s): Oona Liedes
Last updated by author(s): Oct 5, 2025

Reporting Summary

Nature Portfolio wishes to improve the reproducibility of the work that we publish. This form provides structure for consistency and transparency in reporting. For further information on Nature Portfolio policies, see our Editorial Policies and the Editorial Policy Checklist.

Statistics

For all statistical analyses, confirm that the following items are present in the figure legend, table legend, main text, or Methods section.

n/a	Confirmed	
☐	☒	The exact sample size (*n*) for each experimental group/condition, given as a discrete number and unit of measurement
☐	☒	A statement on whether measurements were taken from distinct samples or whether the same sample was measured repeatedly
☐	☒	The statistical test(s) used AND whether they are one- or two-sided<br>*Only common tests should be described solely by name; describe more complex techniques in the Methods section.*
☒	☐	A description of all covariates tested
☐	☒	A description of any assumptions or corrections, such as tests of normality and adjustment for multiple comparisons
☐	☒	A full description of the statistical parameters including central tendency (e.g. means) or other basic estimates (e.g. regression coefficient) AND variation (e.g. standard deviation) or associated estimates of uncertainty (e.g. confidence intervals)
☐	☒	For null hypothesis testing, the test statistic (e.g. *F*, *t*, *r*) with confidence intervals, effect sizes, degrees of freedom and *P* value noted<br>*Give P values as exact values whenever suitable.*
☒	☐	For Bayesian analysis, information on the choice of priors and Markov chain Monte Carlo settings
☒	☐	For hierarchical and complex designs, identification of the appropriate level for tests and full reporting of outcomes
☐	☒	Estimates of effect sizes (e.g. Cohen's *d*, Pearson's *r*), indicating how they were calculated

Our web collection on statistics for biologists contains articles on many of the points above.

Software and code

Policy information about availability of computer code

Data collection	Data was collected in MS Excel version 2408.
Data analysis	Data analysis was performed using MS Excel version 2408, GraphPad Prism version 10.2.3 and 10.4.1,R version 4.2.1 and FlowJo version 10.10.0.

For manuscripts utilizing custom algorithms or software that are central to the research but not yet described in published literature, software must be made available to editors and reviewers. We strongly encourage code deposition in a community repository (e.g. GitHub). See the Nature Portfolio guidelines for submitting code & software for further information.

Data

Policy information about availability of data

All manuscripts must include a data availability statement. This statement should provide the following information, where applicable:

- Accession codes, unique identifiers, or web links for publicly available datasets
- A description of any restrictions on data availability
- For clinical datasets or third party data, please ensure that the statement adheres to our policy

At the outset of the trial, data-sharing provisions were not included in the informed consent documents signed by participants. In accordance with ethics and institutional policies, we are not authorized to release individual-level or pseudo-anonymized datasets to the public. To protect participant privacy, only de-

identified, aggregated group-level values (without background or individual-level information) are available. These data can be requested from the corresponding author (oona.liedes@thl.fi) and will typically be provided within 2–4 weeks, subject to review for compliance with applicable ethical requirements.

Research involving human participants, their data, or biological material

Policy information about studies with human participants or human data. See also policy information about sex, gender (identity/presentation), and sexual orientation and race, ethnicity and racism.

Reporting on sex and gender	Self-reported gender was not available. Biological sex was inferred from the personal identity code (social security number). However, sex was not used as a variable in the statistical analysis.
Reporting on race, ethnicity, or other socially relevant groupings	Race and ethnicity data were not collected. However, participants' occupations were recorded and reported. No analyses were conducted based on occupation or other socially relevant groupings.
Population characteristics	The inclusion criteria for the study were (1) age of 18-65 years, (2) belonging to the target group of the avian influenza vaccine, (3) intention to accept at least one dose of the avian influenza vaccine, (4) a native speaker of Finnish, Swedish or English, (5) home address in Finland, (6) ability to give samples three weeks after each dose, (7) preferably the ability to also participate in the follow-up samplings, and (8) a written informed consent. The exclusion criteria were any medical contraindications to influenza vaccination and a history of anaphylactic reaction to any of the constituents or trace residues of the vaccine.<br><br>Participation in this study was voluntary, so some degree of self-selection bias is possible. The study cohort was limited to occupationally exposed individuals, which may not represent the general population. These factors may limit generalizability but are unlikely to affect internal validity of the immunogenicity findings.
Recruitment	We invited all registered fur and poultry farmers in the wellbeing services counties of Southern, Central and Northern Ostrobothnia and Kainuu by mail, asking them to forward the invitation to their employees. We approached public sector veterinarians, bird ringers and laboratory workers at the Finnish Food Authority, Finnish Institute for Health and Welfare, Helsinki University Hospital and Diagnostic Center, Turku University Hospital and University of Turku, by sending an information letter of the study, and subsequently an invitation letter to those who expressed their interest to participate in the study.
Ethics oversight	The study was conducted in accordance with the Declaration of Helsinki and was authorized by the Finnish Medicines Agency (Fimea) under EU Clinical Trial number 2023-509178-44-00, following evaluation via the EU Clinical Trial Information System (CTIS).

Note that full information on the approval of the study protocol must also be provided in the manuscript.

Field-specific reporting

Please select the one below that is the best fit for your research. If you are not sure, read the appropriate sections before making your selection.

☒ Life sciences ☐ Behavioural & social sciences ☐ Ecological, evolutionary & environmental sciences

For a reference copy of the document with all sections, see nature.com/documents/nr-reporting-summary-flat.pdf

Life sciences study design

All studies must disclose on these points even when the disclosure is negative.

Sample size	The analysis included 39 adult participants divided into two groups: (1) participants belonging to the target groups for whom the avian influenza vaccine is recommended with no previous influenza (A)H5 vaccination history (n=30) and (2) participants from cohort 1 who have previously received H5 influenza vaccines in 2009, 2011–2012 and/or 2018 (n=9). The targeted sample size of 300 for the study cohort 1 was determined using the sample size formula: n= (Z2 x p(1-p))/E2. The calculation was based on a desired 95% confidence level (Z), an assumed seroprotection rate of 75% (p), and a 5% margin of error (E). The result indicates a minimum sample size of 288 subjects required to accurately estimate the proportion of subjects achieving seroprotection. With this sample size, the lower limit of the 95% confidence interval is ≥70%. The number of participants recruited to the study in 2024 remained significantly lower, which will introduce uncertainty into the seroprotection assessment from this sample.
Data exclusions	Participants who did not provide blood samples at the scheduled study visits were excluded from the analysis. Additionally, individuals over the age of 65 were excluded, in accordance with the study's inclusion criteria. In the AIM assays, samples with less than 10,000 CD3+ cells were excluded from all analyses, and samples with less than 500 circulating T follicular helper (cTfh) CD4+ cells were excluded from cTfh cell analysis.
Replication	MN assay included technical replicates. For FMIA, samples were tested at 1:400 and 1:1600 dilutions in duplicate, with results averaged across four wells. Key experiments were repeated with consistent results, confirming reproducibility. Due to the low number of PBMC samples, there were not enough cells to do replications for the AIM assays. To mitigate this, the AIM assay was optimized beforehand. Positive and negative controls were also included.
Randomization	No medical intervention was used in the study and no randomization was applied; all samples meeting the inclusion criteria were analyzed.

Blinding

Investigators were blinded to the identity of the participants during all immunological analyses. For FMIA and HI assays, the investigators were also blinded to the timing of the sample collection with respect to vaccination (i.e., whether samples were collected before or after vaccination). For microneutralization and cellular immunity assays, samples from different time points of the same individual were analyzed in parallel within the same run to ensure comparability. Therefore, the timing of the samples (pre- vs. post-vaccination) was known to the investigators conducting these analyses.

Reporting for specific materials, systems and methods

We require information from authors about some types of materials, experimental systems and methods used in many studies. Here, indicate whether each material, system or method listed is relevant to your study. If you are not sure if a list item applies to your research, read the appropriate section before selecting a response.

Materials & experimental systems

n/a	Involved in the study	
☐	☒	Antibodies
☐	☒	Eukaryotic cell lines
☒	☐	Palaeontology and archaeology
☒	☐	Animals and other organisms
☐	☒	Clinical data
☒	☐	Dual use research of concern
☒	☐	Plants

Methods

n/a	Involved in the study	
☒	☐	ChIP-seq
☐	☒	Flow cytometry
☒	☐	MRI-based neuroimaging

Antibodies

Antibodies used

HRP-conjugated anti-Influenza A antibody (Medix Biochemica, Cat# 100083) and R-PE-conjugated anti-human IgG Fcγ (Jackson ImmunoResearch, Cat# 109-115-098) were used. Fluorochrome-conjugated antibodies used for cytometry were anti-human CD45 (HI30 clone) conjugated with APC-eFluor780 (Invitrogen/Life Technologies, Cat#.47-0459), Anti-human CD3 (UCHT1 clone) conjugated with eFluor506 (Invitrogen/Life Technologies, Cat#. 69-0038-42), Anti-human CD4 (RPA-T4 clone) conjugated with eFluor450 (Invitrogen/Life Technologies, Cat#. 48-0049-42), Anti-human CD8a (SK1) conjugated with PerCP-eFluor710 (Invitrogen/Life Technologies, Cat#. 46-0087-42), Anti-human CD69 (FN50 clone) conjugated with PE (BD Biosciences , Cat#. 555531), Anti-human CD134 (ACT35 clone) conjugated with PE/Cyanine7 (BioLegend, Cat#. 350012), Anti-human CD137 (4B4-1 clone) conjugated with APC (BioLegend, Cat#. 309810), Anti-human CD45RA (HI100 clone) conjugated with Brilliant Violet 785 (BioLegend, Cat#. 304140), Anti-human CD197 (CCR7) (G043H7 clone) conjugated with PE/Dazzle 594 (BioLegend, Cat#. 353236), Anti-human CD185 (CXCR5) (J252D4 clone) conjugated with Brilliant Violet 605 (BioLegend, Cat#. 356930).

Validation

The antibodies used in this study were validated by manufacturers. Anti-Influenza A 7307 SPTN-5 (Medix Biochemica, Cat. #100083, monoclonal IgG1) https://www.medixbiochemica.com/anti-influenza-a-100083. Anti-Human IgG, Fcγ (Jackson ImmunoResearch, Cat. #109-115-098, R-PE conjugate, polyclonal) https://www.jacksonimmuno.com/catalog/products/109-115-098. Fluorochrome-conjugated antibodies used for cytometry were anti-human CD45 (HI30 clone) conjugated with APC-eFluor780 (Invitrogen/Life Technologies, Cat#.47-0459) https://www.thermofisher.com/antibody/product/CD45-Antibody-clone-HI30-Monoclonal/47-0459-42, Anti-human CD3 (UCHT1 clone) conjugated with eFluor506 (Invitrogen/Life Technologies, Cat#. 69-0038-42) https://www.thermofisher.com/antibody/product/CD3-Antibody-clone-UCHT1-Monoclonal/69-0038-42, Anti-human CD4 (RPA-T4 clone) conjugated with eFluor450 (Invitrogen/Life Technologies, Cat#. 48-0049-42), https://www.thermofisher.com/antibody/product/CD4-Antibody-clone-RPA-T4-Monoclonal/48-0049-42, Anti-human CD8a (SK1) conjugated with PerCP-eFluor710 (Invitrogen/Life Technologies, Cat#. 46-0087-42), https://www.thermofisher.com/antibody/product/CD8a-Antibody-clone-SK1-Monoclonal/46-0087-42, Anti-human CD69 (FN50 clone) conjugated with PE (BD Biosciences , Cat#. 555531), https://www.bdbiosciences.com/en-fi/products/reagents/flow-cytometry-reagents/research-reagents/single-color-antibodies-ruo/pe-mouse-anti-human-cd69.555531?tab=product_details, Anti-human CD134 (ACT35 clone) conjugated with PE/Cyanine7 (BioLegend, Cat#. 350012), https://www.biolegend.com/en-gb/products/pe-cyanine7-anti-human-cd134-ox40-antibody-7234?GroupID=BLG9043, Anti-human CD137 (4B4-1 clone) conjugated with APC (BioLegend, Cat#. 309810), https://www.biolegend.com/en-gb/products/apc-anti-human-cd137-4-1bb-antibody-3910, Anti-human CD45RA (HI100 clone) conjugated with Brilliant Violet 785 (BioLegend, Cat#. 304140), https://www.biolegend.com/en-gb/products/brilliant-violet-785-anti-human-cd45ra-antibody-7972, Anti-human CD197 (CCR7) (G043H7 clone) conjugated with PE/Dazzle 594 (BioLegend, Cat#. 353236), https://www.biolegend.com/en-gb/products/pe-dazzle-594-anti-human-cd197-ccr7-antibody-9811, Anti-human CD185 (CXCR5) (J252D4 clone) conjugated with Brilliant Violet 605 (BioLegend, Cat#. 356930), https://www.biolegend.com/en-gb/products/brilliant-violet-605-anti-human-cd185-cxcr5-antibody-12362.

Eukaryotic cell lines

Policy information about cell lines and Sex and Gender in Research

Cell line source(s)

MDCK cell lines (ATCC-CCL-34 and ATCC-CRL-2935) were obtained from the American Type Culture Collection (ATCC).

Authentication

Authentication was performed by the supplier.

Mycoplasma contamination

Tested and negative for Mycoplasma.

Commonly misidentified lines (See ICLAC register) | No misidentified lines were used.

Clinical data

Policy information about clinical studies

All manuscripts should comply with the ICMJE guidelines for publication of clinical research and a completed CONSORT checklist must be included with all submissions.

Clinical trial registration | The study was registered in the EU Clinical Trials Information System (CTIS) under the EU CT number 2023-509178-44-00 on 19 April 2024.

Study protocol | The study was conducted in accordance with the standards of Good Clinical Practice, the Declaration of Helsinki and local legal and regulatory requirements. The full study protocol is available in the EU Clinical Trials Information System (CTIS) as part of the trial registration (EU CT number: 2023-509178-44-00). The study has received authorization from the Finnish Medicines Agency Fimea. Written informed consent to participate was obtained from all participants before sampling. Participation in the study was voluntary and uncompensated.

Data collection | Participants were recruited in Finland starting in June 2024. Recruitment targeted individuals for whom the MF59-adjuvanted A(H5N8) influenza vaccine (clade 2.3.4.4b A/Astrakhan/3212/2020, Seqirus) was recommended—specifically those at risk of exposure to infected animals, including fur and poultry farm workers, veterinarians, bird ringers, and laboratory personnel handling avian influenza viruses or potentially contaminated samples. Sample collection was conducted at laboratory centers within the well-being services counties. Participants were asked to indicate their professional affiliation or vaccine target group.

Outcomes | Primary outcome measure was seroconversion proportion after the second vaccine dose. Secondary outcomes were pre-defined in the study protocol and registered in EU Clinical Trial Registry (2023-509178-44-00).

Plants

Seed stocks | *Report on the source of all seed stocks or other plant material used. If applicable, state the seed stock centre and catalogue number. If plant specimens were collected from the field, describe the collection location, date and sampling procedures.*

Novel plant genotypes | *Describe the methods by which all novel plant genotypes were produced. This includes those generated by transgenic approaches, gene editing, chemical/radiation-based mutagenesis and hybridization. For transgenic lines, describe the transformation method, the number of independent lines analyzed and the generation upon which experiments were performed. For gene-edited lines, describe the editor used, the endogenous sequence targeted for editing, the targeting guide RNA sequence (if applicable) and how the editor was applied.*

Authentication | *Describe any authentication procedures for each seed stock used or novel genotype generated. Describe any experiments used to assess the effect of a mutation and, where applicable, how potential secondary effects (e.g. second site T-DNA insertions, mosiacism, off-target gene editing) were examined.*

Flow Cytometry

Plots

Confirm that:

☒ The axis labels state the marker and fluorochrome used (e.g. CD4-FITC).

☒ The axis scales are clearly visible. Include numbers along axes only for bottom left plot of group (a 'group' is an analysis of identical markers).

☒ All plots are contour plots with outliers or pseudocolor plots.

☒ A numerical value for number of cells or percentage (with statistics) is provided.

Methodology

Sample preparation | Sample preparation is detailed in the 'Activation Induced Marker (AIM) Assay and Flow Cytometry' subsection of the 'Materials and Methods' section.

Instrument | LSRFortessa (BD Bioscience)

Software | FlowJo 10.10.0

Cell population abundance | Cells were seeded at 1,000,000 cells per well, with a minimum of 10,000 $CD3^+$ T cells and over 500 circulating T follicular helper (cTfh) cells required for downstream analysis.

Gating strategy | Gating strategy is shown in the Extended Data Figure 2.

☒ Tick this box to confirm that a figure exemplifying the gating strategy is provided in the Supplementary Information.
